# Fetal and Neonatal Complications Associated With Acute Fatty Liver of Pregnancy

**DOI:** 10.7759/cureus.101976

**Published:** 2026-01-21

**Authors:** Shumaila J Ahmed, Sahar Mudassar, Sana Ali, Narmin Faryal, Tatheer Hasan, Samiha Samad

**Affiliations:** 1 Obstetrics and Gynaecology, Dow University of Health Sciences, Karachi, PAK; 2 Obstetrics and Gynaecology, Civil Hospital Karachi, Karachi, PAK; 3 Family Medicine, Dow University of Health Sciences, Karachi, PAK; 4 Obstetrics and Gynaecology, AEI Telehealth, Toronto, CAN; 5 Obstetrics and Gynaecology, Sohar Hospital, Sohar, OMN

**Keywords:** coagulopathy, fatty liver of pregnancy, liver dysfunction, maternal predictors, neonatal outcome, perinatal mortality

## Abstract

Background

Acute fatty liver of pregnancy (AFLP) is a rare but life-threatening obstetric emergency that can quickly endanger both the mother and fetus, typically presenting in the third trimester. Although greater awareness and supportive management have improved maternal survival, perinatal outcomes remain poor, particularly in resource-limited settings. This study aimed to describe fetal and neonatal complications associated with AFLP and identify maternal factors contributing to poor perinatal outcomes.

Methods

This retrospective study analysed medical records of 73 women diagnosed with AFLP at the Department of Obstetrics and Gynaecology, Unit I, Dr Ruth K. M. Pfau Civil Hospital Karachi, Dow University of Health Sciences, from November 3, 2024, to April 3, 2025. Diagnosis was based on the Swansea criteria. Maternal demographics, presenting symptoms, laboratory parameters, and neonatal outcomes were reviewed. Poor perinatal outcome was defined as stillbirth, early neonatal death, or a neonatal intensive care unit (NICU) stay exceeding seven days. Multivariate logistic regression was used to identify maternal predictors of adverse outcomes.

Results

The mean maternal age was 29 years, with most women presenting at an average gestational age of 35 weeks. Nausea and vomiting were reported in 64 (87.7%), malaise in 56 (76.7%), and jaundice in 47 (64.4%). Coagulopathy occurred in 31 (42.5%), while acute kidney injury was noted in 17 (23.3%). There were 13 stillbirths (17.8%) and five early neonatal deaths (6.8%), resulting in a perinatal mortality of 18 (24.6%). Lower gestational age, coagulopathy, and higher maternal bilirubin levels were independently associated with adverse perinatal outcomes.

Conclusion

Despite improvements in maternal management, perinatal mortality in AFLP remains alarmingly high. Early recognition and timely delivery, ideally before the onset of severe hepatic dysfunction, are critical for improving fetal survival. Strengthening neonatal intensive care facilities, developing national AFLP registries, and establishing standardised management protocols can play a key role in enhancing both maternal and neonatal outcomes in Pakistan and similar settings.

## Introduction

Acute fatty liver of pregnancy (AFLP) is an uncommon, severe, and potentially fatal disease specific to the gestational age, which presents with microvesicular fatty accumulation in the hepatocytes culminating in acute hepatic failure with multiorgan dysfunction [1]. The disease nearly always manifests in the third trimester or shortly after delivery and therefore becomes a valid obstetric and medical emergency. The worldwide incidence varies from one in 7,000 to one in 15,000 pregnancies; however, the syndrome remains vastly underdiagnosed in resource-poor areas due to common clinical presentation and restricted diagnostic potential [2,3].

The pathophysiology of AFLP has been attributed to inherited defects in mitochondrial fatty acid oxidation, most notably fetal long-chain 3-hydroxyacyl-CoA dehydrogenase (LCHAD) deficiency. The defect precipitates the accumulation in the maternal circulation of the harmful fatty acid metabolites, which cause hepatocellular damage, defective β-oxidation, and progressive/systemic dysfunction. The maternal-fetal metabolic interface in this disease also accounts for the reason why AFLP commonly presents with fetal metabolic derangements and perinatal morbidity and mortality [4].

Clinically, the patient experiences an array of nonspecific symptoms, including nausea, vomiting, malaise, right upper quadrant pain, and jaundice. Its clinical presentation can readily resemble other hepatic disease conditions in the context of pregnancy, like preeclampsia, HELLP (haemolysis, elevated liver enzymes, and low platelets) syndrome, or viral hepatitis [5]. Laboratory findings typically show elevated transaminases, elevated bilirubin, hypoglycemia, coagulopathy, and renal insufficiency. Owing to its polymorphic presentation, diagnosis relies on the combination of clinical and biochemical characteristics, which are mainly defined by the Swansea criteria and are helpful in differential diagnosis from other hepatic disease conditions in the progress of pregnancy [6].

In the past two decades, maternal mortality due to AFLP has significantly reduced from nearly 70% in the 1970s to less than 10% in the developed world due to improved recognition, early detection, and initiation of delivery at the earliest if the disease becomes a suspect. Even then, the perinatal mortality rates are still too high at 15-25% globally and up to 25-35% in South Asia and sub-Saharan Africa [7].

In the Pakistani context, literature available for AFLP is scarce and largely from single-centre retrospective studies [8]. Data from tertiary hospitals in Lahore and Karachi report perinatal mortality rates ranging from 26% to 32%, a state largely caused by late presentation, inefficient referral processes, and unavailability of neonatal intensive care units. In contrast, high-income nations, like the United Kingdom [9] and China, have reported studies with perinatal mortality rates of around 18-20%, also demonstrating the advantage of early diagnosis and superior neonatal support [10,11].

Given these disparities, there is a pressing need for region-specific evidence to identify maternal clinical and biochemical markers capable of predicting adverse fetal outcomes in order to intervene early. It was therefore with the aim of (1) evaluating complications in the fetal and neonatal domains associated with AFLP, (2) determining independent maternal perinatal adverse outcome predictors, and (3) comparing these findings with the respective national and worldwide findings in order to guide future clinical practice and policy making.

## Materials and methods

Study design and setting

We conducted an observational retrospective study in the Department of Obstetrics and Gynaecology, Unit-I, Dr. Ruth K. M. Pfau Civil Hospital Karachi, affiliated with the Dow University of Health Sciences (DUHS), Karachi, Pakistan, for a period of six months from November 3, 2024, to April 3, 2025. This is a tertiary care hospital and one of the largest public-sector hospitals, not only serving people of Karachi but also patients who visit for quality treatment from all over Pakistan because of its well-equipped high-dependency obstetric units (HDU) and neonatal intensive care unit (NICU) required for the better treatment of patients with pregnancy and newborn-related complications.

Study population

All pregnant patients diagnosed with AFLP who were managed during the study period in Obstetrics and Gynaecology Unit-I were included. The diagnosis of AFLP was made using the Swansea criteria [12], a set of widely accepted clinical criteria for AFLP in the absence of pathological confirmation. According to the Swansea criteria, at least six of these features listed in Table 1 must be present for a diagnosis of AFLP.

**Table 1:** Swansea criteria, a set of diagnostic criteria used to identify acute fatty liver of pregnancy. Table 1 is independently created by the authors using the stated criteria and is our own original compilation. [12]

No.	Clinical/laboratory feature	Diagnostic threshold/description
1	Persistent vomiting	Recurrent or intractable nausea and vomiting not attributable to other causes
2	Abdominal pain	Right upper quadrant or epigastric pain
3	Polydipsia/polyuria	Excessive thirst and urination suggestive of diabetes insipidus–like symptoms
4	Encephalopathy	Altered level of consciousness or confusion
5	Elevated serum bilirubin	>14 µmol/L
6	Hypoglycemia	Blood glucose <4 mmol/L
7	Elevated serum uric acid	>340 µmol/L
8	Leukocytosis	White blood cell count >11 × 10^9^/L
9	Ascites or bright liver on ultrasound	Evidence of ascites or increased hepatic echogenicity
10	Elevated aminotransferases	ALT or AST >42 IU/L
11	Coagulopathy	Prothrombin time (PT) >14 seconds or INR >1.5
12	Renal impairment	Serum creatinine >1.5 mg/dL

Women with alternate hepatic conditions such as viral hepatitis (A, B, C, E), HELLP syndrome, intrahepatic cholestasis of pregnancy, preeclampsia with severe features, or drug-induced hepatic injury were excluded to maintain diagnostic specificity. In the end, 73 patients were eligible and included in the final analysis. All patients were treated under a multidisciplinary care team of obstetricians, hepatologists, anaesthetists, intensivists, and neonatologists according to institutional clinical management policy for AFLP, which entails stabilisation, reversal of coagulopathy, and prompt delivery once diagnosis is confirmed.

Data collection

Data was collected from patient charts, hospital electronic medical records, and laboratory databases in a formatted template especially constructed for the study. Two research assistants, who were supervised by the principal investigator for the capture of complete and accurate data, recorded all abstracted information.

Maternal demographic and obstetric variables, including age, parity, gravidity, gestational age at the time of diagnosis of AVH, booking status at a health facility, and history of any previous obstetric complications, were documented. Presenting complaints (nausea, vomiting, malaise, jaundice, right upper quadrant pain, altered sensorium) and physical findings (hepatomegaly {oedema}/ascites) along with clinical data were recorded. Mode of delivery (spontaneous vaginal, induction of labour, or caesarean) was documented as well as indication for delivery and maternal complications/concerns such as coagulopathy, disseminated intravascular coagulation (DIC), acute kidney injury, hepatic encephalopathy, and postpartum haemorrhage.

Laboratory examinations were available for all these patients. These included haemoglobin, total leucocyte count (TLC), platelet count, serum bilirubin, alanine aminotransferase (ALT), aspartate aminotransferase (AST), prothrombin time (PT) and international normalised ratio (INR), fluid balance at right heart catheterisation (RHC) day 7-10, serum creatinine, uric acid, blood sugar, and serum albumin. In patients who had several investigations during their stay, the most abnormal result was included in the analysis for disease severity. Hepatic echogenicity, signs of fatty infiltration, or the presence of ascites-based ultrasonographic findings were taken from radiology reports as well.

Information was collected from labour and washing, and NICU folders about the fetal and neonatal baby. This was composed of gestational age at delivery, birthweight, Apgar scores, fetal growth restriction (FGR), neonatal conditions (respiratory distress, hypoglycemia, meconium aspiration, and sepsis), admission to NICU, and discharge. The adverse perinatal outcome included stillbirths and early neonatal deaths (within seven days of life).

Cross-referencing with labour and NICU logs was done to verify data. For any missing or incomplete data, we excluded these patients from the relevant sub-analysis but retained them in descriptive summaries, not to lose their representation. Data obtained from the database were de-identified and coded to ensure patient confidentiality.

Standard obstetric and neonatal terminology was applied according to World Health Organization (WHO) and American College of Obstetricians and Gynecologists (ACOG) definitions to ensure consistency and comparability across clinical categories, including high-risk pregnancy and modes of delivery [13,14]. Preterm birth is when a baby is born prior to 37 weeks of gestation, and low birth weight (LBW) was defined as newborns weighing less than 2.5 kg at birth. Diagnosis of FGR was based on the estimated fetal weight (EFW) or abdominal circumference being below the 10th percentile, meaning it's smaller than 90% of babies at the same stage. Coagulopathy was characterised as a prothrombin time more than five seconds above control or an INR > 1.5. Hypoglycemia was defined as an RbGl <70 mg/dL.

The composite outcome was an adverse perinatal outcome consisting of stillbirth, early neonatal death (death of a neonate in the first seven days), or NICU admission ≥7 days. This composite endpoint was created to represent the entire spectrum of fetal and neonatal morbidity and mortality resulting from AFLP.

Data analysis

Statistical analysis was carried out with IBM SPSS Statistics for Windows, version 25.0 (IBM Corp., Armonk, NY, USA). Data entered was cross-checked for accuracy, and normality of continuous variables was assessed with the Kolmogorov-Smirnov test. Descriptive statistics were performed and displayed for all variables: continuous as mean ± standard deviation (SD) and categorical as frequencies and percentages. Emergency Department use during the year 2015 was calculated.

We also compared women who had favourable and adverse perinatal outcomes to detect possible risk factors. For continuous variables, the independent-sample t-test (normal distribution) or Mann-Whitney U test (non-normal distribution) was selected. Associations between categorical variables, including both coagulopathy and maternal complications, as well as NICU admission, were examined using chi-square tests or Fisher’s exact tests.

Multinomial logistic regression analysis was performed to find out independent predictors for adverse perinatal outcomes. Factors with a p < 0.10 in univariate analysis were included in the multivariate model. The predictor variables were adjusted odds ratio (aOR) and 95% CI for predictors of the adverse outcome.

Goodness of fit for the model was examined using the Hosmer-Lemeshow test and receiver operating characteristic (ROC) curve analysis for the purpose of discriminating ability evaluation through the area under the ROC curve (AUC). An AUC ≥0.80 was defined as strong discrimination. The model’s explanatory power was presented as Nagelkerke R². All statistical tests were two-tailed, and a p-value of <0.05 was considered significant.

Ethical considerations

This study was approved by the Institutional Review Board (IRB) of Dow University of Health Sciences, Karachi (IRB-3395/DUHS/Approval/2023/1329), prior to data collection. Because this was a retrospective study utilising anonymised hospital records, the requirement for informed consent was formally waived by the IRB. Confidentiality and privacy were maintained at every stage of the research. Each patient’s identifiable information was removed before data entry, and electronic data were stored on password-protected devices accessible only to the research investigators.

No direct patient interaction or intervention was undertaken, and the study posed no risk to the participants. All ethical procedures conformed to the principles outlined in the Declaration of Helsinki and the International Committee of Medical Journal Editors (ICMJE) recommendations for ethical publication of medical research.

## Results

Baseline and clinical characteristics

The mean maternal age was 29.1 ± 5.4 years, and the mean gestational age was 35.3 ± 2.2 weeks. Nausea/vomiting (87.7%), malaise (76.7%), jaundice (64.4%), and abdominal pain (56.2%) were common presenting symptoms. Most deliveries occurred via emergency caesarean section (82.2%) (Table 2).

**Table 2:** Baseline and clinical characteristics (n = 73)

Variable	Mean ± SD	n (%)
Maternal age (years)	29.1 ± 5.4	-
Gestational age (weeks)	35.3 ± 2.2	-
Primigravida	-	40 (54.8%)
Caesarean section	-	60 (82.2%)
Coagulopathy	-	31 (42.5%)
Acute kidney injury	-	17 (23.3%)
Postpartum haemorrhage	-	7 (9.6%)

The findings show that anaemia was present in 100% of patients (mean haemoglobin 9.9 ± 1.3 g/dL), while thrombocytopenia occurred in 100% (mean platelet count 105 ± 36 × 10^9^/L). Hyperbilirubinaemia was observed in 100% (mean bilirubin 5.3 ± 3.1 mg/dL), and marked transaminase elevation (ALT 301 ± 116 IU/L, AST 317 ± 128 IU/L) was also seen in all cases. Prolonged PT/INR, indicating coagulopathy, was noted in 100%, and elevated creatinine, suggesting renal impairment, occurred in approximately 90-100% of patients. Overall, nearly all patients demonstrated multi-organ involvement consistent with acute liver failure or severe hepatic dysfunction, such as AFLP or HELLP syndrome (Table 3).

**Table 3:** Haematological and biochemical parameters ALT: alanine aminotransferase; AST: aspartate aminotransferase; PT: prothrombin time; INR: international normalised ratio

Parameter	Mean ± SD	Normal range
Haemoglobin (g/dL)	9.9 ± 1.3	11.5–15.0
Platelet count (×10^9^/L)	105 ± 36	150–400
Bilirubin (mg/dL)	5.3 ± 3.1	<1.2
ALT (IU/L)	301 ± 116	<35
AST (IU/L)	317 ± 128	<35
PT (s)	24.3 ± 5.9	11–16
INR	2.0 ± 0.4	<1.2
Creatinine (mg/dL)	1.7 ± 0.8	0.5–1.0

The comparison between the two groups shows that Group B had a significantly lower mean gestational age (33.6±1.8 weeks) compared to Group A (35.8±2.0 weeks, p = 0.013). The frequency of coagulopathy was also higher in Group B (56.0%) than in Group A (35.4%), which was statistically significant (p = 0.021). In addition, serum bilirubin and ALT levels were significantly elevated in Group B (6.9±3.7 mg/dL and 341±119 IU/L, respectively) compared to Group A (4.8±3.0 mg/dL and 278±102 IU/L), with p-values of 0.041 and 0.049, respectively. These findings indicate that Group B patients had more severe hepatic dysfunction and coagulopathy, along with earlier gestational age at presentation (Table 4).

**Table 4:** Comparison between favourable and adverse perinatal outcomes (n = 73) Group A represents cases with favourable perinatal outcomes, while Group B represents cases with adverse perinatal outcomes (stillbirth, early neonatal death, or NICU stay ≥ 7 days). ALT: alanine aminotransferase; NICU: neonanatal intesive care unit *A p-value <0.5 is statistically significant.

Variable	Group A (n = 48)	Group B (n = 25)	Test used	Test statistic value	p-value
Gestational age (weeks)	35.8 ± 2.0	33.6 ± 1.8	t-test	t = 2.54	0.013*
Coagulopathy present	17 (35.4%)	14 (56.0%)	χ² test	χ² = 5.32	0.021*
Serum bilirubin (mg/dL)	4.8 ± 3.0	6.9 ± 3.7	t-test	t = 2.10	0.041*
ALT (IU/L)	278 ± 102	341 ± 119	t-test	t = 2.01	0.049*

Perinatal outcomes

Among the 73 pregnancies, there were 13 stillbirths (17.8%) and five early neonatal deaths (6.8%), resulting in a perinatal mortality of 24.6%. Of the live births, 61.6% were preterm, and 58.9% required NICU admission. Multivariate analysis showed that lower gestational age was significantly associated with adverse outcomes (aOR = 0.62, 95% CI 0.45-0.88, p = 0.007). Maternal coagulopathy independently increased the risk (aOR = 4.35, 95% CI 1.11-17.08, p = 0.035), while higher total serum bilirubin levels were also a significant predictor (aOR = 1.27, 95% CI 1.01-1.59, p = 0.043). These results indicate that earlier gestational age, presence of maternal coagulopathy, and elevated neonatal total bilirubin are strong predictors of poor outcomes (Table 5).

**Table 5:** Independent predictors of adverse outcomes (n = 73) aOR: adjusted odds ratio; AUC: area under the curve Model: Multivariable logistic regression model adjusted for clinically relevant maternal and obstetric covariates.

Predictor	aOR	95% CI	p-value
Gestational age (per week)	0.62	0.45–0.88	0.007
Coagulopathy	4.35	1.11–17.08	0.035
Serum total bilirubin (per mg/dL)	1.27	1.01–1.59	0.043

**Figure 1:**
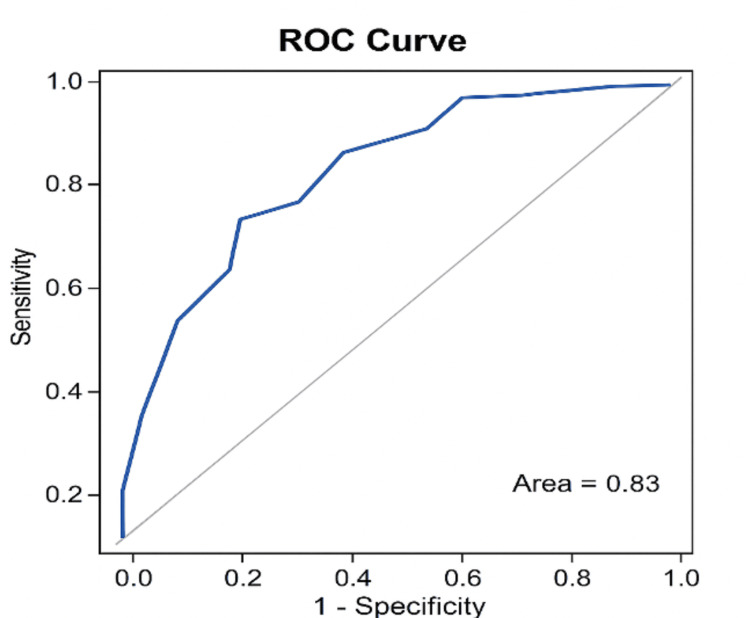
Receiver operating characteristic (ROC) curve of the predictive model

A ROC curve analysis demonstrated that the multivariable logistic regression model had good discriminative ability for predicting adverse perinatal outcomes. The AUC was 0.83, indicating that the model correctly distinguished between favourable and adverse outcomes 83% of the time. This level of discrimination reflects strong predictive performance, suggesting that gestational age, coagulopathy, and maternal serum bilirubin collectively provide meaningful prognostic information in AFLP (Figure 1).

## Discussion

In a six-month, single-centre study that included 73 women with a diagnosis of AFLP, the perinatal mortality was noted to be 24.6%, and preterm birth (PTB) in 61.6%, as well as NICU admissions in 58.9%. Our results are generally consistent with earlier national and international data and document important contextual differences that can account for such outcome variability [15]. In Pakistan, the described perinatal mortality in AFLP is higher, between 26% and 32%, based on a study of tertiary care hospitals from Lahore, Karachi, and Peshawar [8]. The relatively lower death rate may even be indicative of the recent surge in medical practices at the tertiary level presented at Dr Ruth K. M. Pfau Civil Hospital due to early detection, stabilisation, and expeditious delivery. However, our findings show that despite the advances in maternal survival, perinatal outcomes are still not optimal when compared with high-income settings, where the perinatal mortality rates were noted as 15-20% [16].

The small survival benefit we noted in our institution is probably associated with earlier obstetric decision-making and better blood product availability for correction of coagulopathy prior to delivery. Nonetheless, the higher rate of NICU admission in these neonates reflects ongoing risks associated with being born to mothers with AFLP, among whom prematurity and metabolic abnormalities are prevalent. When compared with international cohorts, we found that coagulopathy and high bilirubin were stronger predictors of adverse perinatal outcome, while parameters like renal dysfunction or encephalopathy had less impact in our population, which are well-known predictors in the Western data [17,18]. This distinction is most likely due to the timing and stage of presentation. In developed countries, early diagnosis by biochemical screening in asymptomatic infants prevents hepatic synthetic failure, whereas in low-income country reports like our own, cases usually present late with more severe hepatic dysfunction, hence marked maternal coagulopathy and neonate's hyperbilirubinemia at presentation. Therefore, these two variables become useful early predictors of perinatal compromise in our setting [19].

The relationship between small-for-gestation at birth and poor neonatal outcome observed in our study has also been reported worldwide [20]. Survival improves with each added week of gestation due to improved pulmonary maturity, glucose control, and thermoregulation. Immediate management of AFLP at the time of delivery not only saves the mother’s life in many cases but may also save the neonate's life, though it imparts a risk of prematurity to the fetus if delivery is before 35 weeks. This clinical compromise helps to explain why the gestational age of a fetus continues to be an important determinant of perinatal outcome. The high correlation of maternal coagulopathy with adverse neonatal outcome underscores the systemic nature of AFLP. Not only is it a reflection of severe hepatic dysfunction, but coagulopathy also causes or aggravates uteroplacental hypoperfusion and fetal hypoxia. In addition, treatment of coagulopathy might postpone parturition and prolong exposure of the fetus to a threatening intrauterine environment. Furthermore, hyperbilirubinaemia also represents a biochemical marker of liver injury and cholestasis, which, in turn, correlates with the severity of disease [21,22]. In our study, any rise in bilirubin level was associated with a significant odds ratio for fetal distress and early neonatal death, similar to findings in reports from China [23] and South Korea [24], where excess maternal serum bilirubin appeared to be associated with placental hypoxia and fetal compromise.

Our results are also in line with international statistics on neonatal morbidities. The most common complications were preterm delivery, respiratory distress, and hypoglycemia, demonstrating intrauterine stress as well as the immaturity of neonatal metabolic adjustment. Neonatal outcomes have been reported in studies from Tanzania [25], China [26], and Zivaljevic et al. [27], where most of their neonates receiving ventilation survived with no abnormal long-term consequences for survivors, demonstrating a higher level of NICU care capacity, including surfactant availability and advanced mechanical ventilation, compared to their local NICUs. The higher neonatal mortality in Pakistani cohorts, including ours, is therefore mostly due to lack of facilities for postnatal care and not so much because of disease biology. This highlights the importance of improving neonatal intensive care, ensuring access to respiratory support/surfactant/glucose monitoring is available, and educating clinicians on the management of premature infants born following maternal hepatic crisis [28].

The mode of birth is also a factor that influences the outcome. The caesarean section (CS) rate of our study was 82.2%, which reflects the available recommendations that support early surgical delivery after maternal decompensation or fetal distress. Caesarean delivery reduces exposure to the toxic intrauterine environment and improves maternal stabilisation, but does not completely eliminate risks for the neonate, particularly in early gestations. The aim, therefore, should not be a uniform increase in CS rates but rather a timely transfer and delivery prior to maternal decompensation. The problem in Pakistan is that there is delayed presentation at the peripheral hospitals followed by delayed referral to the tertiary centres, where it can be safely managed, and sometimes patients come to tertiary care centres late when they are already in advanced stages of this disease [29].

Compared to global AFLP literature, our study supports some overarching trends and provides context-specific exceptions. The fundamental factors contributing to adverse neonatal outcomes persist: prematurity, hepatic insufficiency, and coagulopathy. They are, however, exaggerated in settings of limited resources, lack of immediate recognition, and poor neonatal care. Early recognition of AFLP with a strategy for biochemical screening programs in the developed countries, combined with tertiary NICU care, has reduced perinatal mortality to 15-18% [30]. South Asian and African publications, however, still present higher numbers, reflecting the gaps in timely access to obstetric and neonatal interventions [31,32]. Our results, therefore, contribute to the emerging regional literature showing that the gap is not one of knowledge but of speed of diagnosis and access to supportive care.

From a global perspective, our study supports other findings that maternal and perinatal outcomes in AFLP rely on timing and available resources more than on inherent differences in disease power. To improve outcomes in Pakistan, a three-tier approach, including the following, will be important: early recognition at the district level by awareness of alarm signs and basic laboratory screening; referral with early stabilisation for correction of coagulopathy; and an expansion of NICU capacity to care for preterm and metabolically unstable neonates. The international experience shows that integrated obstetric-neonatal protocols and programmatic management can reduce maternal and perinatal mortality by as much as 50%. We will thus need to translate our findings into practice not through new clinical but rather system-level discoveries, i.e., improving laboratory turnaround time, blood product availability or neonatal care infrastructure, and better referring such cases in a tertiary care hospital

Limitations and recommendations

There are a few limitations in this study that should be corrected in future studies. As a single-centre retrospective study, potential documentation and case selection biases are present. Tissue screens were not conducted (e.g., LCHAD), and long-term follow-up of surviving neonates was not feasible. To counter these limitations, develop a national AFLP registry for the accumulation of multicentre data. Implement routine liver and coagulation function screening in symptomatic women >32 weeks of gestation, ensure NICU with ventilatory and metabolic support capabilities, initiate newborn metabolic testing to detect LCHAD deficiency early in the newborn period, and encourage multidisciplinary education between obstetric, hepatic, and neonatal teams.

## Conclusions

In this series of 73 pregnancies complicated by AFLP, perinatal mortality remained high (24.6%), with prematurity, maternal coagulopathy, and raised bilirubin as independent predictors of adverse outcome. While maternal survival is getting better, neonatal outcomes are worse than those observed with early diagnosis and with appropriate infrastructure for neonates readily available. Strengthening obstetric screening programs, timing of delivery, and neonatal critical care are all important measures to decrease perinatal mortality in Pakistan and improve its performance towards the international norms.
